# Correlates of substance abuse treatment completion among disadvantaged communities in Cape Town, South Africa

**DOI:** 10.1186/1747-597X-5-3

**Published:** 2010-03-11

**Authors:** Bronwyn J Myers, Sonja Pasche, Mohamed Adam

**Affiliations:** 1 Alcohol and Drug Abuse Research Unit, South African Medical Research Council, PO Box 19070, Tygerberg, 7505, South Africa; 2 Department of Psychiatry and Mental Health, University of Cape Town, Private Bag X1, Rondebosch, 7705, South Africa; 3 Department of Psychology, University of the Western Cape, Private Bag X17, Bellville, 7535, South Africa

## Abstract

**Background:**

Completion of substance abuse treatment is a proximal indicator of positive treatment outcomes. To design interventions to improve outcomes, it is therefore important to unpack the factors contributing to treatment completion. To date, substance abuse research has not examined the factors associated with treatment completion among poor, disadvantaged communities in developing countries. This study aimed to address this gap by exploring client-level factors associated with treatment completion among poor communities in South Africa.

**Methods:**

Secondary data analysis was conducted on cross-sectional survey data collected from 434 persons residing in poor communities in Cape Town, South Africa who had accessed substance abuse treatment in 2006.

**Results:**

Multiple regression analyses revealed that therapeutic alliance, treatment perceptions, abstinence-specific social support, and depression were significant partial predictors of treatment completion.

**Conclusions:**

Findings suggest that treatment completion rates of individuals from poor South African communities can be enhanced by i) improving perceptions of substance abuse treatment through introducing quality improvement initiatives into substance abuse services, ii) strengthening clients' abstinence-oriented social networks and, iii) strengthening the counselor-client therapeutic alliance.

## Background

In recent years, substance-related problems have increased in South Africa [1], with the lifetime prevalence rate for substance use disorders estimated to be around 13% [2]. Compared to other parts of the country, substance-related problems are particularly prevalent in Cape Town, the capital of the Western Cape Province [2]. Despite high levels of treatment need, the availability of substance abuse treatment facilities is limited in this region, particularly among poor disadvantaged Black African and Coloured communities [3,4] (where "Coloured" refers to a cultural grouping of mixed race ancestry). In these communities, substance-related problems are prevalent and barriers to substance abuse treatment use (such as limited availability of affordable services) abound [4].

In the light of these widespread barriers, it is vital that service providers optimize treatment outcomes for individuals from poor communities who manage to access services. One strategy for increasing the likelihood of positive outcomes is to ensure individuals complete their allotted treatment episode as treatment completion has been consistently associated with positive outcomes [5-8]. For this to occur, factors associated with completion of substance abuse treatment need to be identified. Although studies conducted in the USA have identified several client-level factors associated with completion of substance abuse treatment (such as the therapeutic alliance [9], perceptions of and satisfaction with treatment [10,11], treatment motivation [8,12], and social support for treatment [13]), the extent to which these findings are applicable to developing countries such as South Africa is unknown. This negatively impacts on service providers' ability to design evidence-based interventions to improve completion rates, and consequently substance abuse treatment outcomes. This study aims to redress this gap through exploring client-level variables associated with completion of substance abuse treatment for persons from poor communities in Cape Town, South Africa.

## Methods

### Sample characteristics

The sample consisted of 434 individuals from disadvantaged communities in Cape Town who accessed substance abuse treatment in the 12 months prior to the study. To be included in the study, subjects had to be at least 18 years of age, earn less than ZAR 2500 per month (at the time of the study USD 1 = ZAR 8.50), self-identify as Black African or Coloured, reside in one of the study's 12 target communities, have a substance use disorder as classified by the DSM-IV-TR [14], and have accessed treatment within the 12 months preceding the study. Subjects were recruited from a range of disadvantaged communities. Specifically, two residential areas from each of the six sub-structures of Cape Town were selected as key focus areas. To be selected, the area had to consistently appear in the South African Community Epidemiology Network on Drug Use's list of top ten residential areas for substance abuse problems. Selected areas also had to be classified as a "Black African" or "Coloured" residential area under the apartheid regime (as these areas remain relatively more disadvantaged than areas previously classified for white South Africans only), have high levels of health and social problems, and be a low-income area. The sample comprised almost equal proportions of male (*n *= 236) and female (*n *= 198), as well as Black African (*n *= 221) and Coloured (*n *= 213) individuals.

### Research and sampling procedure

Secondary data analysis was conducted on unanalyzed cross-sectional survey data collected by the South African Medical Research Council's (MRC) Alcohol and Drug Abuse Research Unit between June 2006 and January 2007. This survey examined factors associated with substance abuse treatment utilization for persons from poor, disadvantaged communities.

As treatment utilization among individuals from poor disadvantaged communities in Cape Town is a rare event and there are no population-level registers of individuals who have received substance abuse treatment in South Africa from which random sampling could have been conducted, snowball sampling was used to identify potential participants. Non-profit treatment centres offering specialised substance abuse treatment programmes were identified as starting points for sampling, as compared to the for-profit treatment sector, they are more likely to serve clients from poor communities. All of these services offered structured substance abuse treatment programmes and employed health and social welfare professionals to deliver their programmes. At the time of the study, these non-profit treatment facilities comprised twelve residential short and medium stay programmes ranging in duration from 21 days to three months, eight outpatient programmes of varying intensity and duration (n = 12), two specialised hospital programmes, and one detoxification programme.

Counsellors from these facilities were trained to identify possible subjects. Contact information for these potential recruits was passed onto a team of five fieldworkers, with the written consent of the recruits. These fieldworkers were responsible for collecting survey data from the participants. All fieldworkers had extensive experience in conducting community surveys related to substance use, were familiar with the target recruitment areas, and had completed 40 hours of training in data collection procedures. Fieldworkers contacted recruits to conduct a structured interview that took approximately 90 minutes to complete. These recruits in turn referred the data collectors to other potential recruits until the desired sample size had been obtained. Response bias was minimised by obtaining a response rate of 98%. Recall bias was limited by using time-line follow back (TLFB) procedures to collect retrospective data [15]. Ethical approval for the original study and the secondary data analyses was granted by the Ethics Review Board of the Faculty of Humanities at the University of Cape Town and the Faculty of Community and Health Sciences at the University of the Western Cape respectively.

### Measures

Although the original study collected self-report data on a range of variables thought to be associated with substance abuse treatment use, this study only examined treatment completion and factors thought to be associated with this criterion variable. Participants were asked to described their substance abuse treatment history and name the various treatment programmes they had attended. Past 12-month use of treatment and the type of treatment received (residential, outpatient or hospital programme) was examined. Completion of last substance abuse treatment episode was assessed by the question: "The last time you were in substance abuse treatment, did you complete the treatment programme?" This item had a "yes" (1) or a "no" (0) response. This self-report data on treatment completion was validated by the counsellors responsible for referring the participant to the research team.

The following demographic variables were examined: age, gender, race, and years of education. Psychological functioning was examined using the Texas Christian University (TCU)'s 6-item depression and 7-item anxiety scales. For these scales, higher scores indicate greater levels of depression and anxiety [16]. This study obtained an alpha co-efficient of .92 for each scale.

Motivation for treatment was measured using the TCU's three motivation scales: the 9-item problem recognition (PR) scale, a 6-item desire for help (DH) scale and the 8-item treatment readiness (TR) scale. For these scales, higher scores indicate greater motivation [16]. The Cronbach alpha co-efficients obtained were sufficiently high, ranging from .68 to .97 across the three scales. Abstinence-specific social support was assessed using the TCU's 9-item social support scale which measures the extent to which one's social network supports abstinence from substance use and engagement in treatment [16]. Higher scores indicate more abstinence-specific support. An alpha co-efficient of .77 was obtained for this scale.

Clients' perceptions of treatment were measured using the 10-item Treatment Perceptions Questionnaire (TPQ), with higher scores on this questionnaire indicating more positive perceptions of treatment [11]. This study obtained an alpha co-efficient of .93 for the TPQ. Therapeutic alliance was measured by a 13-item Counselling Rapport scale. This scale measures constructs such as the collaborative and affective bond between client and therapist. Higher scores on this scale indicate a stronger alliance [16]. The alpha co-efficient obtained for this scale was .97.

Prior to implementing the main study, these measures were pilot-tested among 40 people with substance-related problems from two disadvantaged communities in Cape Town in order to establish the cultural validity of these measures. Pilot-testing took the form of face-to face interviews. This one-on-one interview format allowed researchers to obtain feedback on the extent to which interview items were understood and to identify problematic items that needed to be changed. Items were generally well-understood and few problems arose. Negatively worded items were changed due to misunderstandings that occurred, and examples and probing questions were added to ensure that the meaning behind the items would be understood and to minimise the occurrence of "don't know" and "neutral" responses. Pilot-testing also allowed the reliability of the scales to be established for a South African population.

### Analytic approach

Bivariate analyses were conducted to examine the differences between completers and non-completers of treatment. As the data did not fit a normal distribution, the Mann-Whitney *U *test was used to examine continuous data, while the association between categorical variables and completion were examined using Chi-square analyses. Variables that were significantly associated with completion in bivariate analyses were then entered into a logistic regression model using a forward stepwise procedure. This procedure was performed to determine the variables associated with treatment completion when controlling for the influence of the other variables.

## Results

### Description of completers and non-completers

Of the 434 individuals recruited into the study, 298 (69%) had completed their previous treatment episode. Of the people who completed treatment, 40.5% were female and 59.5% were male. Similarly, 53.1% of non-completers were male and 46.9% were female (Table 1). In terms of race, 53.7% of treatment completers were Black African and 46.3% were Coloured. The average age of completers and non-completers was similar (25.28 and 24.47 years old respectively; Table 1). Completers and non-completers also had similar mean years of education, levels of employment, and types of drug problems (Table 1).

**Table 1:** Demographic characteristics of sample and type of treatment received

Variable		Completers	Non-completers
**Demographic**			
Mean age in years (SD)		25.28 (4.73)	24.47 (4.89)
Age first started using drugs			
Mean years of education (SD)		11.45 (1.64)	11.69 (1.45)
% Employed		51.4	55.4
Race	% Black African	53.7	46.9
	% Coloured	46.3	53.1
Gender	% male	59.5	53.1
	% female	40.5	46.9
Type of drug problem*			
% Alcohol		29.9	32.7
% Cannabis		59.9	66.5
% Methaqualone		32.8	31.9
% Crack/cocaine		16.4	16.3
% Methamphetamine		38.4	34.2
% Heroin		16.4	12.8
**Total (N)**		**298**	**136**

*Does not add up to 100% as some have polysubstance problems

All participants received specialised substance abuse treatment services. In this sample, 61.3% of participants received speciality outpatient services, 33.6% received residential services, 3.9% received a hospital-based substance abuse treatment programme, and 1.4% received detoxification only. Data were not collected on the model of treatment or intensity of care provided by these various programmes. In terms of treatment completion, 55.62% of participants who received outpatient services completed their treatment programme and 81.47% of participants who attended residential services completed their programme.

### Bivariate analyses

Demographic variables that significantly differentiated between treatment completers and non-completers included age and age of first drug use. Older individuals and those who initiated drug use at an older age were more likely to complete treatment than younger participants (Table 2). Gender [χ^2 ^(1, N = 434) = 1.53, p = .25] and race [χ^2 ^(1, N = 434) = 0.78, p = .41] failed to differentiate between completers and non-completers.

**Table 2:** Mann-Whitney tests: Comparisons of completers and non-completers

Variable	Completers		Non-completers		Mann-Whitney test	
	*Mdn (Interquartile range)*	*Mean rank*	*Mdn (Interquartile range)*	*Mean rank*	*U*	*p*
**Demographic**						
Age in years	25.00 (22.00-29.00)	226.84	24.00 (20.00-27.00)	197.03	17479.5	.010*
Age first started using drugs	18.00 (16.00-20.00)	225.67	17.00 (16.00-19.00)	199.61	17830.5	.040*
Years of education	12.00 (11.00-13.00)	215.65	11.00 (11.00-13.00)	221.55	19713.0	.635
**Other factors**						
Counseling Rapport	43.00 (38.00-51.00)	255.29	36.00 (30.00-41.50)	134.69	9001.5	.0001***
Treatment Perceptions	32.00 (30.00-35.00)	253.90	24.00 (22.00-28.00)	137.74	9416.0	.0001***
Anxiety	40.00 (40.00-44.29)	233.14	40.00 (34.29-40.00)	183.22	15602.5	.0001***
Treatment Readiness	32.50 (30.00-37.50)	231.72	30.00 (30.00-35.00)	186.34	16026.0	.0001***
Social Support	37.78 (35.56-40.00)	231.65	37.77 (33.33-37.77)	186.50	16047.5	.0001***
Problem Recognition	37.78 (35.56-41.11)	230.18	37.78 (33.89-37.78)	189.72	16486.0	.001**
Desire for Help	40.00 (37.14-42.86)	229.61	38.57 (34.29-40.00)	190.97	16656.0	.001**
Depression	40.00 (36.67-41.67)	228.46	40.00 (31.66-40.00)	193.48	16997.0	.006**
**Total (N)**	**298**		**136**			

* *p *< .05; ** *p *< .01; ** *p *< .001

The two variables most strongly associated with treatment completion were therapeutic alliance and treatment perceptions, with an increase in either of these variables significantly improving the chances of successfully completing treatment (Table 2). Other variables that significantly differentiated between completion and non-completion were the treatment motivation scales, abstinence-specific social support and the psychological functioning scales (anxiety and depression); with an increase in any of these scales improving the likelihood of completion (Table 2).

### Multivariate analyses

Logistic regression was used to examine the predictors of treatment completion. A test of the full model versus the model with the intercept only was significant (χ^2^(4) = 140.83; *p *< .001); with the model predicting 39% of the estimated variance in completion (Nagelkerke *R*^2 ^= .39). According to the Hosmer and Lemeshow test, the model adequately fitted the data (χ^2^(8) = 11.32; *p *= .184). In the final model, treatment perceptions, therapeutic alliance, abstinence-specific social support and depression had significant partial effects on treatment completion (Table 3). Age, age of first drug use, anxiety and the three treatment motivation scales did not enter the model. This model was able to classify 80.5% of participants who completed treatment and 54.2% of participants who did not complete treatment correctly.

**Table 3:** Logistic regression: Significant predictors of treatment completion

Predictor variables	B *(SE)*	Wald (df)	Odds Ratio (95% CI)
Depression	0.07 (0.02)	20.45 (1)***	1.08 (1.04 -- 1.11)
Treatment perceptions	0.11 (0.03)	18.45 (1)***	1.12 (1.06 -- 1.18)
Therapeutic alliance	0.08 (0.02)	9.54 (1)**	1.08 (1.03 -- 1.13)
Social support	0.08 (0.03)	7.09(1)**	1.08 (1.02 -- 1.14)

* *p *< .05; ** *p *< .01; *** *p *< .001; Nagelkerke *R*^2 ^= .39

## Discussion

Findings from this study are potentially useful for the design of interventions to improve substance abuse treatment outcomes for people from disadvantaged communities who have limited access to substance abuse services, both in South Africa and other developing countries. Although substance abuse treatment completion is known to be a proximal indicator of more distal treatment outcomes [6-8], efforts to improve substance abuse treatment in developing countries have been hampered by a lack of local, contextual knowledge on factors associated with completion of treatment. This study begins to address this gap by unpacking some of the client-level variables associated with completion of substance abuse treatment for people from disadvantaged communities in Cape Town, South Africa.

Our findings show that the factor most strongly associated with treatment completion among people from disadvantaged communities in Cape Town, South Africa, was perceptions of and satisfaction with substance abuse treatment services. Specifically, the likelihood of treatment completion increased as perceptions of treatment and satisfaction with services improved. The important role that perceptions of treatment play in the process of substance abuse treatment is confirmed by earlier findings that negative perceptions of treatment also act as a barrier to substance abuse treatment entry among persons from disadvantaged South African communities [17]. The positive association of treatment perceptions with treatment completion is consistent with findings from studies conducted in more developed country settings [5,10] which highlight the contribution that positive perceptions of care makes to level of engagement in and the completion of substance abuse treatment. This finding has important implications for the improvement of substance abuse treatment services in South Africa. One means of improving clients' perceptions of treatment (and thereby treatment completion rates) would be to introduce service quality improvement initiatives into publicly-funded treatment services. This would require some shifts in current substance abuse treatment policy. In general, the focus of substance abuse treatment policy and service planning in the country has been to improve the availability of services [4] and the quality of available services has been a neglected aspect of service planning and policy [4,18]. In addition to improving service availability, policy makers and service planners therefore should focus on improving the quality of existing services. A first step to improving service quality would be to monitor the performance of treatment services on key performance indicators and use findings to implement interventions aimed at improving service performance [18].

Our findings also point to other client-level factors associated with treatment completion. The positive associations of therapeutic alliance and abstinence-specific social support with treatment completion are consistent with findings from studies conducted in developed country settings [9,12,13]. While motivation for treatment was significantly associated with completion of treatment in bivariate analyses, it was not associated with completion in multivariate analyses. These findings suggest that motivation has an indirect effect on treatment completion. Earlier studies have suggested that motivation has an indirect effect on completion via the therapeutic alliance [12], however the extent to which this is explanation is applicable to the South African context requires additional investigation. In contrast, our finding of an association between greater levels of psychological distress and longer retention conflicts with findings from previous studies which found no evidence of such an association [19,20]. It is possible that individuals may enter, remain engaged in and complete treatment in an attempt to alleviate psychological distress. This explanation however, requires further investigation.

Taken together, these findings have important implications for interventions to improve rates of treatment completion in developing countries. In many developing countries, confrontational styles of treatment that counter the formation of a strong therapeutic alliance are widely used. Counselors in these settings thus require training in treatment styles that enhance the therapeutic alliance, and subsequently treatment retention. In addition, service providers should facilitate the development of abstinence-oriented social networks through involving supportive family and friends in the treatment process as well as through encouraging clients to participate in twelve-step support groups [13]. Also, service providers should consider implementing systems that allow for the ongoing monitoring of clients' progress in and perceptions of treatment. This is important as perceptions of treatment (including treatment satisfaction) act as a warning signal for potential dropout from treatment [21]. Monitoring treatment perceptions thus could allow service providers to make program adjustments that prevent attrition from treatment.

Despite these implications, findings from this study should be considered in the light of several limitations. First, the use of a cross-sectional design precluded a temporal examination of the factors associated with treatment completion and thus inferences about causality could not be drawn. Second, as this study sample was limited to adults from poor disadvantaged communities in the Cape Town region, the extent to which findings from this study are generalizable to the broader South African population are questionable. Related to this, as our sample was drawn almost entirely from counseling referrals, it is possible that counselors may have inadvertently introduced a selection bias into our sample by systematically excluding potential participants from our study who may have been suitable to include and/or including recruits who were model clients. Third, our model (based on client-level variables) was much better at classifying people who completed treatment than those who did not complete treatment. This implies that we may have excluded important variables from the analysis that could account for why people from disadvantaged communities drop out of treatment. As the study sample comprised individuals from poor communities, it is possible that structural barriers (such as the costs of treatment and transport difficulties) effected participants' ability to complete treatment [4]. Future studies should therefore include a focus on structural influences on treatment completion. In addition, longitudinal prospective studies that track clients in substance abuse treatment services and allow researchers to unpack the factors that precipitate treatment completion would address many of the limitations of the current study. These future studies also would benefit from collecting data on the proportion of treatment seekers who manage to access and complete treatment relative to those who seek services and are unable to obtain them as well as on characteristics of the treatment organization (including styles and types of treatment programs provided) as such data will hold important implications for treatment service development in the country [22].

## Conclusions

Despite some limitations this study provides good preliminary evidence that client-level variables, such as perceptions of treatment, therapeutic alliance and abstinence-specific social support, play an important role in the completion of substance abuse treatment for persons from poor communities in Cape Town, South Africa. Findings point to various improvements that can be made to substance abuse treatment policy and service delivery in the country. First, perceptions of existing treatment services need to be improved through service quality improvement initiatives. This would require, as a first step, shifts in existing treatment policy to include an expanded focus on service quality and effectiveness. The introduction of performance monitoring systems into South African substance abuse treatment services would also provide a solid foundation for evidence-based performance improvement enterprises. Efforts to improve the therapeutic alliance and clients' perceptions of treatment as well as expand clients' abstinence-specific social support networks also may yield positive benefits for rates of treatment completion. Finally, researchers should consider conducting experimental studies to examine whether efforts to improve these client-level variables positively affect rates of substance abuse treatment completion.

## Competing interests

The authors declare that they have no competing interests.

## Authors' contributions

BM conceived the study, participated in its design and coordination, and helped draft the manuscript; SP participated in the design and coordination of the study, performed the statistical analysis, and helped draft the manuscript; MA participated in the design and coordination of the study and helped draft the manuscript. All authors read and approved the final manuscript.
